# Delivery Rate Affects Uptake of a Fluorescent Glucose Analog in Murine Metastatic Breast Cancer

**DOI:** 10.1371/journal.pone.0076524

**Published:** 2013-10-18

**Authors:** Narasimhan Rajaram, Amy E. Frees, Andrew N. Fontanella, Jim Zhong, Katherine Hansen, Mark W. Dewhirst, Nirmala Ramanujam

**Affiliations:** 1 Department of Biomedical Engineering, Duke University, Durham, North Carolina, United States of America; 2 Duke University School of Medicine, Duke University, Durham, North Carolina, United States of America; 3 Department of Radiation Oncology, Duke University, Durham, North Carolina, United States of America; Tufts University, United States of America

## Abstract

We demonstrate an optical strategy using intravital microscopy of dorsal skin flap window chamber models to image glucose uptake and vascular oxygenation *in vivo*. Glucose uptake was imaged using a fluorescent glucose analog, 2-[N-(7-nitrobenz-2-oxa-1,3-diaxol-4-yl)amino]-2-deoxyglucose (2-NBDG). SO_2_ was imaged using the differential absorption properties of oxygenated [HbO_2_] and deoxygenated hemoglobin [dHb]. This study was carried out on two sibling murine mammary adenocarcinoma lines, 4T1 and 4T07. 2-NBDG uptake in the 4T1 tumors was lowest when rates of delivery and clearance were lowest, indicating perfusion-limited uptake in poorly oxygenated tumor regions. For increasing rates of delivery that were still lower than the glucose consumption rate (as measured *in vitro*), both 2-NBDG uptake and the clearance rate from the tumor increased. When the rate of delivery of 2-NBDG exceeded the glucose consumption rate, 2-NBDG uptake decreased with any further increase in rate of delivery, but the clearance rate continued to increase. This inflection point was not observed in the 4T07 tumors due to an absence of low delivery rates close to the glucose consumption rate. In the 4T07 tumors, 2-NBDG uptake increased with increasing rates of delivery at low rates of clearance. Our results demonstrate that 2-NBDG uptake in tumors is influenced by the rates of delivery and clearance of the tracer. The rates of delivery and clearance are, in turn, dependent on vascular oxygenation of the tumors. Knowledge of the kinetics of tracer uptake as well as vascular oxygenation is essential to make an informed assessment of glucose demand of a tumor.

## Introduction

In spite of heterogeneities in causal molecular events and signaling pathways, almost all cancers exhibit enhanced glucose uptake relative to normal cells. This enhanced glucose uptake has been utilized clinically for identifying cancers using Positron Emission Tomography (PET) of Fluorodeoxy-glucose (FDG), a radiolabeled analog of glucose. FDG-PET has found widespread use in identifying and staging cancers, and in predicting response to therapy [1]–[5]. PET images are interpreted based on the Standardized Uptake Value (SUV) of FDG. Although computationally simple, this method only provides a snapshot of the kinetics of glucose uptake and clearance, measuring FDG uptake at a single time point approximately 60 minutes after injection [6]. Knowledge of the initial kinetics of tracer uptake is important because blood flow rates and hence the delivery of FDG can influence tracer uptake [7]. FDG does not provide information regarding tumor blood flow or oxygenation and this typically requires the injection of additional tracers. Knowledge of tracer kinetics assumes added importance in the case of predicting response to therapy because therapies such as radiation can cause changes to tumor vasculature, potentially affecting blood flow in addition to expected changes in tumor glycolytic demand.

Here, we demonstrate an optical strategy using intravital microscopy of dorsal skin flap window chamber tumor models to image glucose uptake and vascular oxygenation (SO_2_) *in vivo*, and examine the effects of delivery and decay kinetics on glucose uptake. Intravital microscopy of window chamber models provides the requisite spatial resolution to differentiate vascular from tissue/tumor compartments. Glucose uptake was imaged using a fluorescent analog of FDG, 2-[N-(7-nitrobenz-2-oxa-1,3-diaxol-4-yl)amino]-2-deoxyglucose (2-NBDG). 2-NBDG has been validated in multiple model systems by our group and others as a viable marker of glucose uptake [8]–[14]. Optical imaging is amenable to repeated measures to capture the delivery and clearance kinetics of glucose uptake via 2-NBDG fluorescence. SO_2_ was imaged using the differential absorption properties of oxygenated and deoxygenated hemoglobin. SO_2_ in blood vessels is strongly associated with tissue pO_2_ [15]. Our group has previously established the ability to image SO_2_ of hemoglobin in blood vessels using label-free trans-illumination microscopy in window chamber models [16].

This study was carried out on two sibling murine mammary adenocarcinoma lines, 4T1 and 4T07, derived from the same parental line. These cell lines were selected due to differences in their metastatic potential, glycolytic phenotypes and oxygen consumption properties [17]–[20]. First, the glucose uptake rate of both cell lines was characterized *in vitro*. Second, *in vivo* tumors were established for each cell line. Their baseline vascular and metabolic characteristics were imaged using intravital microscopy. Third, a hypoxia/reoxygenation protocol was used to perturb delivery of the tracer to the tumor. The purpose of this protocol was to perform continuous imaging of SO_2_ and 2-NBDG in response to a reoxygenation-associated increase in blood flow. Two endpoints were obtained from trans-illumination microscopy: concentrations of oxy-hemoglobin ([HbO_2_]) and deoxy-hemoglobin ([Hb]), from which SO_2_ ([HbO_2_]/[Hb]+[HbO_2_]) was derived. Three endpoints were obtained from the 2-NBDG fluorescence kinetic profile: rate of delivery (ratio of the maximum 2-NBDG intensity and the time to maximum), rate of clearance (rate of decay of 2-NBDG intensity from its maximal value to that at 60 minutes), and finally, uptake of 2-NBDG by the tumor after wash-in and wash-out through the vasculature at approximately 60 minutes.

Our results initially revealed a simple relationship between SO_2_ and 2-NBDG uptake. The 4T07 tumors were better oxygenated than the 4T1 tumors and mean 2-NBDG uptake was significantly higher in the 4T1 tumors. Breathing hypoxic gas significantly increased SO_2_ and blood flow in the 4T1 tumors and decreased mean 2-NBDG uptake in the 4T1 tumors to the level of the 4T07 tumors. Detailed analysis revealed that both 4T1 and 4T07 tumors demonstrated distinct patterns of 2-NBDG uptake that depended on the rates of uptake and clearance of 2-NBDG that were, in turn, dependent on tumor SO_2_. The results presented in this manuscript establish the importance of tracer kinetics and SO_2_ in order to accurately interpret glucose uptake data from tumors *in vivo*.

## Methods

### Ethics Statement

This study was carried out in accordance with the recommendations in the Guide for the Care and Use of Laboratory Animals of the National Institutes of Health. The protocol was approved by the Duke University Institutional Animal Care and Use Committee (Protocol Number: A170-12-06). All experiments were performed under isoflurane gas anesthesia, and all efforts were made to minimize suffering.

### *In vitro* Cell Culture

A 4T1 murine mammary carcinoma line was transduced by retroviral siRNA to constitutively express the red fluorescent protein (RFP) DsRed, allowing easy demarcation and growth tracking of tumor cells both *in vitro* and *in vivo* [21]. 4T1-RFP and 4T07 cell lines were cultured in Dulbecco’s Modified Eagle Medium (DMEM) supplemented with 10% fetal bovine serum (FBS) and 1% antibiotics and plated for 24 hours for *in vitro* experiments. After 24 hours, 3 cell plates of each cell line were incubated with 2-NBDG for increasing durations ranging from 1–75 minutes (Incubation time periods were 1, 2, 3, 4, 5, 10, 20, 30, 40, 50 and 75 minutes). For each incubation period, cells were washed once with PBS and incubated with 3 ml of 100 µM 2-NBDG dissolved in glucose-free and serum free-media. At the end of incubation, cells were washed with PBS and imaged immediately using a two photon microscope. 2-NBDG used in these experiments was synthesized and characterized at the Duke University Small Molecule Facility.

### Two Photon Imaging of Cells

2-NBDG fluorescence in the cells was excited at 960 nm and imaged over the wavelengths 495–540 nm. 960 nm was selected to reduce contribution from fluorescence of flavin adenine dinucleotide (FAD). The image size was 512×512 pixels and this corresponded to a field of view of 510×510 µm. Dwell time for each pixel was 8 µs and total image acquisition time was 8.31 s. From each image, the 10 brightest cells were selected to compute the mean fluorescence intensity. Fluorescence images were calibrated using a rhodamine solution (90.8 µM) using the same microscope settings.

### *In vivo* Studies

**Figure 1:**
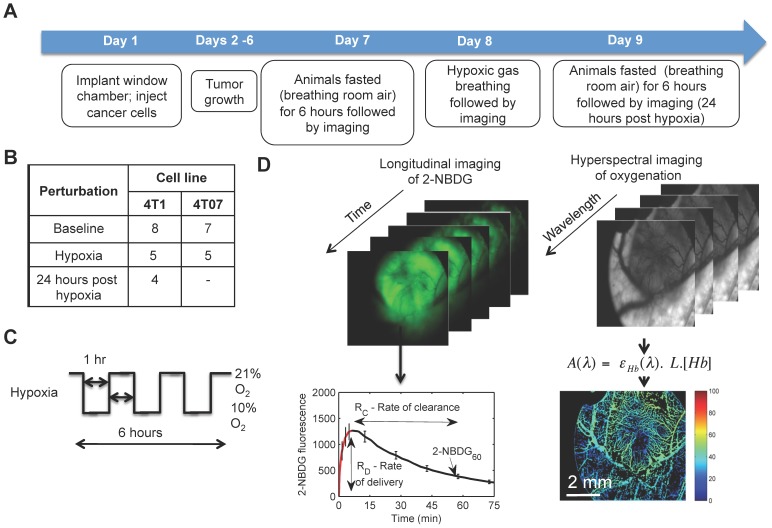
Methods. **A.** Flowchart describing study design. **B.** Sample size for the study. **C.** Illustration of hypoxia protocol. **D.** 2-NBDG fluorescence images are acquired continuously for a period of 75 minutes to construct a (x,y,λ) data cube. B) At each (x,y) pixel location, a time course of 2-NBDG uptake can be obtained. Based on the time course, three metabolic parameters can be calculated: the initial rate of delivery (R_D_), rate of clearance (R_C_), and glucose uptake (2-NBDG_60_). C) Trans-illumination image cube of hemoglobin absorption is obtained from 520–620 nm. Total hemoglobin content is calculated pixel-wise by fitting a Beer-Lambert equation to the hyperspectral dataset. Knowledge of the wavelength-dependent extinction coefficients of hemoglobin allows calculation of oxy-hemoglobin and deoxy-hemoglobin concentrations. The ratio of oxy-hemoglobin to total hemoglobin concentration is the vascular oxygenation (SO_2_).

8 to 10 weeks old mice weighing between 20 and 25 g were used for these studies. Titanium window chambers were surgically implanted on the back of female athymic nude mice (nu/nu, NCI, Frederic, Maryland) under anesthesia (i.p. administration of ketamine (100 mg/kg) and xylazine (10 mg/kg)). A 20 µL suspension (20,000 cells) of 4T1-RFP or 4T07 cells was injected into the dorsal skin fold and a glass coverslip (dia = 12 mm, No. 2, Erie Scientific, Portsmouth, New Hampshire) was placed over the exposed tissue. All animals were housed in an on-site housing facility with *ad libitum* access to food and water and standard 12-hour light/dark cycles. A flowchart depicting the experiment protocol is presented in **Figure 1**. For baseline measurements, the animals were kept in a chamber filled with 21% oxygen for 6 hours. For hypoxia, the animals were exposed to alternating 1-hour cycles of 21% oxygen and 10% oxygen for 6 hours as described below. During this 6-hour period, the animals were only provided water.

### Hypoxia Chamber

The hypoxia chamber used for the *in vivo* experiments was developed in our lab. The chamber was designed using a 37″×37″×25″ Glove Bag (Glas-Col, Terre Haute, IN). The bag included an outlet opening to which a flexible tubing piece 1 cm in diameter with a 6 mm bore was attached. An inlet hole was also made with a #10 scalpel blade to accommodate another piece of identical tubing. The area around each hole was sealed with Blenderm (3 M, St. Paul, MN). The inlet tubing was used to connect to either a 10% oxygen tank (AirGas National Welders, Raleigh, NC) or to house air. This inlet tube could also be attached to a vacuum for gas removal. The outlet tube was fed into an Erlenmeyer flask filled with water in the fume hood, and the tip of the tube was placed under 1-cm of water in the flask. This tube acted as an overflow valve for the system and also allowed the user to visually confirm that the bag was receiving adequate gas flow by assessing bubbling in the water. The large opening of the bag was sealed by wrapping the open end around a piece of rigid tubing and sealing with binder clips. Control mice breathed room air and were kept in the same part of the room; therefore, they experienced similar lighting and noise environments to the treatment mice.

### Imaging Platform

We used a Zeiss Axioskop 2 microscope for recording all images. The imaging platform used in our experiments has been described in detail in a previous publication [22]. Briefly, a 100 W halogen lamp was used for trans-illumination imaging and a 100 W mercury lamp was employed for epi-illumination fluorescence imaging of 2-NBDG and RFP expression. Hyperspectral imaging was accomplished using a liquid crystal tunable filter (LCTF). Trans-illumination images were acquired from 520 to 620 nm in 10 nm increments. Epi-illumination 2-NBDG fluorescence images were acquired at 525 nm with a 470 nm bandpass excitation filter (40 nm bandwidth) and a 510 nm longpass dichroic beamsplitter. Image acquisition time was 300 ms. RFP fluorescence was recorded from 610 to 690 nm using a 560 nm bandpass excitation filter (30 nm bandwidth) and a 600 nm longpass dichroic beamsplitter. The image acquisition time was 100 ms. All images were collected with a 2.5×objective (NA = 0.075) and a DVC 1412 CCD camera (DVC Company). Lamp throughput images at each wavelength were collected by placing a neutral density filter (to avoid saturating the camera) in the optical path. A rhodamine solution (90.8 µM) was used to calibrate images and compare to 2-photon microscope images.

### Hyperspectral Imaging of Vascular Oxygenation and Glucose Uptake

After 6 hours of breathing room air or hypoxic gas, mice were anesthetized using isofluorane mixed with 21% oxygen (1.5% v/v) administered through a nose cone. Once breathing was stable, mice were placed on a heating pad for the duration of the experiment. Trans-illumination images, RFP fluorescence (for 4T1-RFP cells) and a background image at 525 nm corresponding to pre-NBDG injection were recorded. Any background fluorescence at this wavelength was due to FAD. 100 µl of 2-NBDG (2.5 mg/ml) dissolved in sterile saline was injected through the tail vein. The 2-NBDG fluorescence was recorded for 75 minutes as follows: continuously for the first 8 minutes, every 30 seconds for the next 30 minutes and every 3 minutes for the final 35 minutes of imaging.

High resolution images of 2-NBDG uptake and constitutive tumor RFP fluorescence were recorded using a Zeiss upright confocal microscope. 2-NBDG fluorescence was excited at 488 nm and imaged from 510–550 nm. RFP fluorescence was recorded from 580–620 nm. All images were collected using a 10×objective (NA = 0.45). Field of view for the first set of images (baseline –40 minutes) was 850×850 µm. After 40 minutes, field of view was 297×297 µm.

### Calculation of Vascular and Metabolic Parameters

A modified form of the Beer-Lambert law that describes absorption of chromophores in thin slices is fit to the trans-illumination image cube (x,y,λ) to obtain the concentration of the primary absorbers – [HbO_2_] and [dHb] at each pixel [16]. This is possible due to knowledge of the extinction coefficients of both absorbers. Based on this information, we can calculate total hemoglobin content, [THb] ([HbO_2_]+[dHb]) and SO_2_ ([HbO_2_]/[THb]) at each pixel. Because [THb] is negligible in tissue space (hemoglobin exists in blood vessels only), [THb] was used to segment the blood vessels and create a map of SO_2_.

2-NBDG fluorescence images were acquired continuously for a period of 75 minutes to construct a (x,y,λ) data cube. At each (x,y) pixel location, a time course of 2-NBDG uptake was obtained. Based on the time course, three metabolic parameters were calculated at each pixel (Fig. 1): the initial rate of delivery (R_D_), rate of clearance (R_C_), and glucose uptake (2-NBDG_60_). R_D_ was calculated from the rising part of the curve as (I_max_-I_0_)/T_max_, where subscript 0 corresponds to a pre-2-NBDG baseline image. R_C_ was calculated from the falling part of the curve as (I_max_-I_60_)/(T_60_-T_max_). 2-NBDG_60_ is defined as the glucose uptake by the tumor after delivery and clearance from the extracellular space.

### Statistical Analysis

*In vitro* glucose consumption rates were compared using an unpaired Student’s t-test. Statistical analyses of the effects of hypoxia/reoxygenation were performed using a paired Student’s t-test. Correlations between parameters were evaluated by computing Spearman’s rank correlation coefficient (ρ). Survival curves or cumulative probability distributions were compared using repeated measures ANOVA. All statistical analyses were performed using the Statistics Toolbox in MATLAB.

## Results

### 4T1 and 4T07 Cells have Similar Glucose Uptake Rates *in vitro*

**Figure 2:**
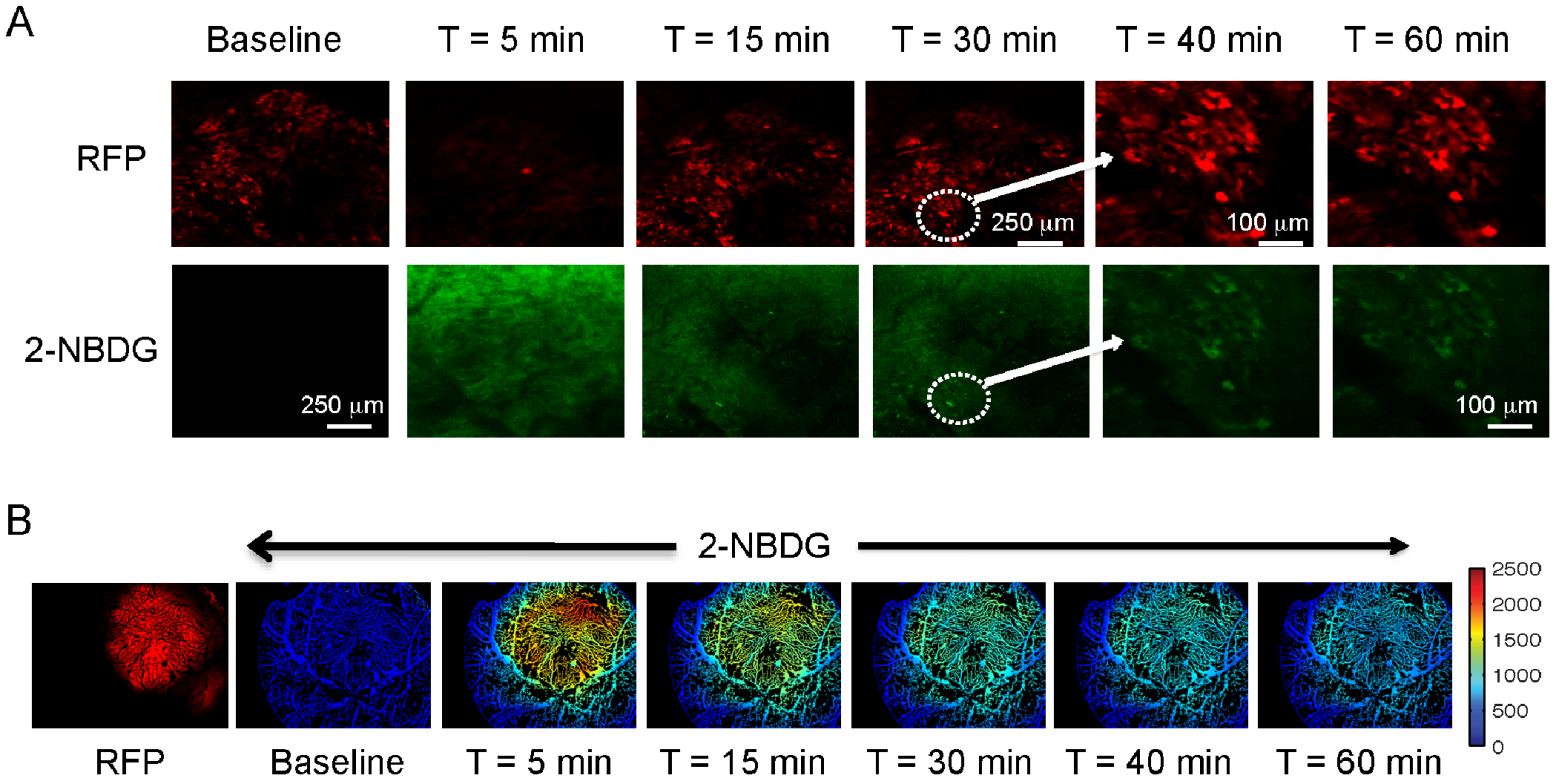
*In vitro uptake* of 2-NBDG in 4T1 and 4T07 murine breast cancer cells. **A.** Representative images from 4T1 and 4T07 cells incubated with 100 µM 2-NBDG for a period of 1–75 minutes. Cell plates were washed after each time point and imaged. **B.** Mean 2-NBDG fluorescence at each time point calculated from the images. Fluorescence values at each time point are normalized to the fluorescence measured at 1 minute. Error bars represent standard error of the mean. Incubation experiments were repeated 3 times for each cell line.

**Figure 2A** presents the dynamics of 2-NBDG-uptake in 4T1 and 4T07 cells *in vitro*. The data show a monotonic increase in 2-NBDG-fluorescence with incubation time. *In vivo*, this is analogous to 2-NBDG being taken up by cancer cells from the interstitial space. The initial rate of glucose uptake was calculated using the 1-minute time point after incubation (**Fig. 2B**). The rate of glucose uptake is similar in both cell lines (2.33±0.09 s^−1^ for 4T1 and 2.69±0.45 s^−1^ for 4T07 cells). Statistical analysis confirmed that the initial rates were not significantly different from each other (p = 0.67).

### 2-NBDG-fluorescence at 60 Minutes after Delivery (2-NBDG_60_) is Indicative of Glucose Uptake within the Tumor *in vivo*

**Figure 3:**
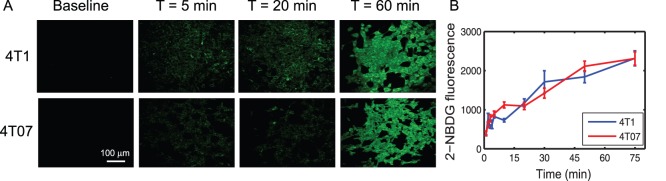
**A. *In vivo uptake* of 2-NBDG in 4T1 dorsal window chamber tumors.** Confocal fluorescence images of a dorsal skin flap window chamber with a 4T1 tumor. Red fluorescence is due to constitutive expression of RFP in 4T1 cells. 2-NBDG images show cellular accumulation and co-registration with RFP at and beyond 40 minutes. Note that first four columns (before 2-NBDG injection, 5, 15, and 30 minutes after injection) are at lower resolution (scale bar is 250 µm) and the third and fourth columns are at higher resolution (scale bar is 100 µm). These images are from the dotted ROI shown in column 2. **B.** 2-NBDG fluorescence images acquired from a 4T1 tumor at lower resolution. The entire tumor is visible in these images. An image of constitutive tumor RFP illustrates the extent of the tumor. Images are acquired at the same time points indicated for 3A. Fluorescence images are not background-subtracted.

To image 2-NBDG *in vivo*, it is necessary to account for the kinetics of 2-NBDG through the vasculature and extracellular space. Thus, a time point for measurement after injection was identified that would be free of the effects of delivery and clearance of 2-NBDG. **Figure 3A** shows confocal fluorescence images of a dorsal window chamber model of a 4T1 tumor. Constitutive RFP expression allows for visualization of the tumor cells at high resolution. There is no significant background fluorescence prior to the injection of 2-NBDG. At 15 minutes after injection, 2-NBDG is present in the entire field of view but is not yet confined to tumor cells. By 30 minutes, 2-NBDG preferentially localizes within the tumor. A population of cells in the lower left corner helps visualize this phenomenon. The fifth and sixth columns present higher resolution images from this region of interest. After 60 minutes, 2-NBDG fluorescence is largely confined to RFP-positive areas. Therefore, 2-NBDG-fluorescence at 60 minutes –2-NBDG_60_– was used to reflect steady state glucose uptake within the tumor *in vivo*.

**Figure 3B** presents 2-NBDG fluorescence images from a tumor acquired using the hyperspectral imaging microscope over a larger field of view. These images are similar to the confocal fluorescence images that exhibit maximum 2-NBDG fluorescence at 5 minutes and a steady decline over the course of 60 minutes.

### Blood Flow and SO_2_ of Tumors Increase after Breathing Hypoxic Gas

**Figure 4:**
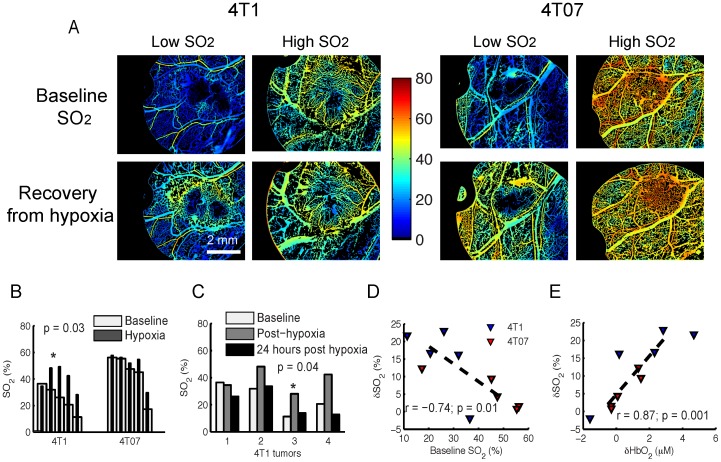
Effect of breathing hypoxic gas on vascular oxygenation of 4T1 and 4T07 tumors. **A.** Representative intravital images of vascular oxygenation (SO_2_) from 4T1 and 4T07 tumors at baseline and after breathing hypoxic gas (10% O_2_, rest N_2_). The effect of hypoxia is compared on tumors that showed low and high SO_2_ values at baseline. White dotted line in each image represents the tumor. **B.** After breathing hypoxic gas, there was a statistically significant increase in SO_2_ of the 4T1 tumors (n = 5; p = 0.03). There was no statistically significant increase in the 4T07 group (n = 5; p = 0.06). Error bars represent standard error of the mean. *indicates statistical significance at alpha = 0.05. **C.** The change in SO_2_ of 4T1 and 4T07 tumors after breathing hypoxic gas is inversely correlated with SO_2_ value at baseline (r = −0.74, p = 0.01). **D.** The change in SO_2_ of 4T1 and 4T07 tumors after hypoxia is strongly correlated with a change in oxy-hemoglobin concentration (r = 0.87; p = 0.001). **E.** 24 hours after breathing hypoxic gas, there was a significant decrease in SO_2_ of the 4T1 tumors (n = 4; p = 0.04), which had returned to a mean value close to pre-hypoxia baseline. 1 animal had to be censored before the 24 hours post-hypoxia measurement. Statistical significance in B and D were assessed using Student’s paired t-test.

**Figure 4** shows representative images of SO_2_ from the 4T1 and 4T07 tumors at baseline and after breathing hypoxic gas. Because both tumor lines exhibited a wide range of SO_2_ values at baseline, representative images from animals with high and low SO_2_ are shown. The 4T1 and 4T07 tumors with low SO_2_ possess a hypoxic core and a negative gradient in SO_2_ towards the center of the tumor. The tumors with high SO_2_ do not exhibit a distinct gradient in SO_2_. After breathing hypoxic gas, the differences between the low and high SO_2_ images at baseline are no longer apparent in either tumor line. Breathing hypoxic gas significantly increased SO_2_ of the 4T1 tumors (**Fig. 4B**; p = 0.03). Although SO_2_ in 4T07 tumors increased after hypoxia, this was not statistically significant (p = 0.06). In the 4T1 tumors, the significant increase in SO_2_ with hypoxic gas breathing was a transient effect with SO_2_ going back to baseline levels 24 hours after the perturbation (**Fig. 4C**; p = 0.04). The increase in SO_2_ was strongly, but negatively associated with baseline SO_2_ in both cell lines (**Fig. 4D**; r = 0.74, p = 0.01). Further, the increase in SO_2_ was driven by an increase in [THb] (data not shown; r = 0.72, p = 0.02) and specifically [HbO_2_] (**Fig. 4E**; r = 0.87, p = 0.001), reflecting increased blood flow in both cell lines after breathing hypoxic gas.

### 2-NBDG_60_ is Dependent on SO_2_ and Hence Perfusion of 4T1 and 4T07 Tumors

**Figure 5:**
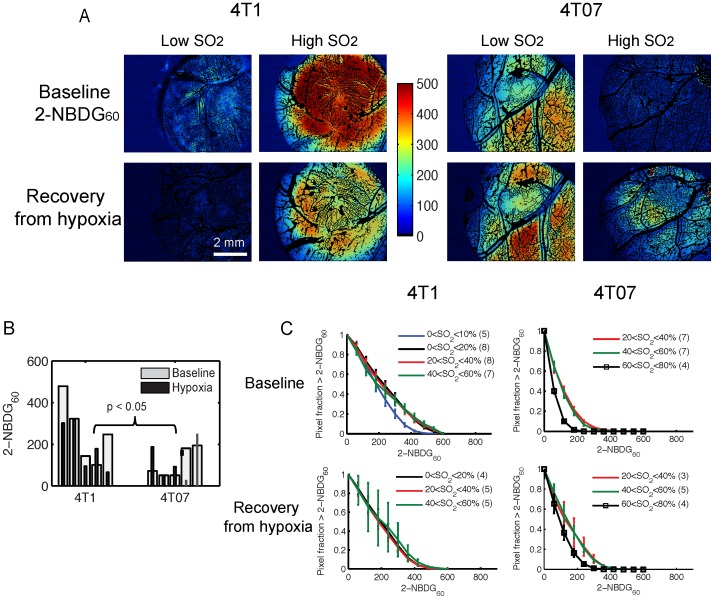
Effect of breathing hypoxic gas on 2-NBDG uptake by 4T1 and 4T07 tumors. **A.** Representative intravital images of 2-NBDG_60_ from 4T1 and 4T07 tumors at baseline and after breathing hypoxic gas (10% O_2_, rest N_2_). 2-NBDG_60_ is the background-subtracted fluorescence image at 60 minutes (I_60_−I_0_). For comparison, 2-NBDG_60_ of tumors with low and high SO_2_ from 4T1 and 4T07 groups are shown here. **B.** 2-NBDG_60_ at baseline is significantly different between the 4T1 and 4T07 tumors. There are no significant changes in mean 2-NBDG_60_ after hypoxia. Each set of bars in 5B represents data from one animal at baseline and after hypoxia (5 animals per cell line). **C.** Survival curves of 2-NBDG_60_ from all animals as a function of SO_2_ of nearest blood vessel. At baseline, 2-NBDG_60_ of 4T1 tumors is lowest when SO_2_<10% (blue line; p<0.01). There are no significant differences in 2-NBDG_60_ for all other SO_2_ ranges. In the 4T07 tumors, 2-NBDG_60_ is lowest when SO_2_>60%. There are no significant differences in 2-NBDG_60_ for other SO_2_ ranges. Only SO_2_ ranges for which there are at least 3 animals are plotted. After breathing hypoxic gas, there are no significant differences in 2-NBDG_60_ between the different SO_2_ ranges in 4T1 and 4T07 tumors, except SO_2_>60% in the 4T07 tumors. Number of animals in each SO_2_ range is shown in parantheses in the legend. Error bars represent standard error of the mean. Statistical tests to compare 2-NBDG_60_ survival curves were conducted using repeated measures ANOVA.

**Figure 5** presents representative 2-NBDG_60_ images of the low and high SO_2_ tumors shown in Fig. 4. At baseline, 2-NBDG_60_ is higher in the high-SO_2_ 4T1 tumor compared to the low-SO_2_ tumor. However, in the 4T07 tumors, 2-NBDG_60_ is lower in the high-SO_2_ tumor. At baseline, mean 2-NBDG_60_ of the 4T1 tumors is significantly higher than the 4T07 tumors (p<0.05). After breathing hypoxic gas, mean 2-NBDG_60_ decreases in the 4T1 tumors and increases in the 4T07 tumors (**Fig. 5B**). However, this is not statistically significant. Mean 2-NBDG_60_ is not significantly different between the two cell lines after hypoxia. There is no crosstalk between SO_2_ and 2-NBDG fluorescence with these measurements because SO_2_ images are acquired prior to 2-NBDG injection. To determine the relationship between SO_2_ and 2-NBDG_60_, survival curves (1-cumulative distributions) of 2-NBDG_60_ for all animals over the entire tumor were plotted as a function of SO_2_ range (**Fig. 5C**). 2-NBDG_60_ in the 4T1 tumors is lowest for the lowest oxygenation level (SO_2_<10%), demonstrating perfusion-limited delivery of 2-NBDG. There are no significant differences in the survival curves of 2-NBDG_60_ at higher SO_2_ levels. Post-hypoxia survival curves show no significant differences in 2-NBDG_60_ as a function of SO_2_. In addition, the lowest SO_2_ range no longer exists after hypoxia due to increased SO_2_. The overall 2-NBDG_60_ is lower post-hypoxia in the 4T1 tumors compared to that at baseline. In the 4T07 tumors, 2-NBDG_60_ is lowest when SO_2_ is highest (60<SO_2_<80%, p = 0.02). There is no significant difference between the other SO_2_ ranges. After breathing hypoxic gas, the same CDF profile is retained in the 4T07 tumors. It is interesting to note that 2-NBDG_60_ is lowest in the 4T1 tumors at lowest SO_2_ and in the 4T07 tumors at highest SO_2_. To understand whether the these results were due to increased blood flow, the kinetic profiles of 2-NBDG uptake and specifically, the effect of increasing SO_2_ on R_D_ was evaluated.

### Increased SO_2_ Leads to Increased Rate of Delivery (R_D_) and Clearance (R_C_) of 2-NBDG

**Figure 6:**
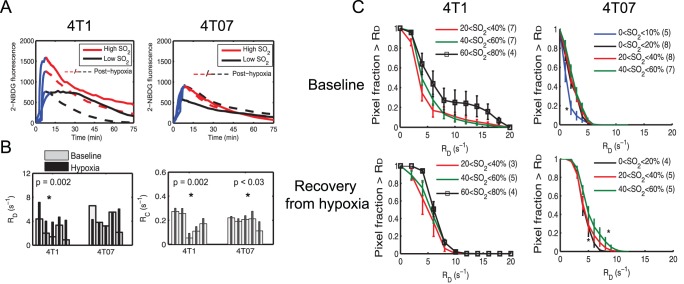
Effect of breathing hypoxic gas on 2-NBDG kinetics. **A.** Kinetic profiles of 2-NBDG uptake in 4T1 and 4T07 tumors for high and low SO_2_. Blue line corresponds to the initial rate of delivery (R_D_). Rate of clearance (R_C_) is calculated from the falling part of the curve. Solid lines indicate baseline data and dashed lines indicate post-hypoxia data. **B.** Breathing hypoxic gas caused a significant increase in R_D_ of 4T1 tumors (p = 0.002). There was significant increase in R_C_ of 4T1 and 4T07 tumors after breathing hypoxic gas. Each set of bars in 6B represents data from one animal at baseline and after hypoxia (5 animals per cell line). **C.** Survival curves illustrating the relationship between R_D_ and SO_2_. In the 4T1 tumors, R_D_ is lowest when SO_2_<10%, illustrating that low SO_2_ leads to poor perfusion. After hypoxia, the region of lowest SO_2_ is no longer present in the 4T1 tumors, indicating an increase in SO_2_. There is a small, but significant increase in R_D_ with an increase in SO_2_. In the 4T07 tumors, R_D_ is highest for the highest level of SO_2_ (>60%). Post hypoxia, there are no significant changes in R_D_ as a function of SO_2_. Statistical tests to compare 2-NBDG_60_ survival curves were conducted using repeated measures ANOVA. For each SO_2_ range, the number of animals is indicated in parentheses. Error bars represent standard error of the mean. *indicates statistical significance at a = 0.05.

**Figure 6A** shows the kinetic profiles of 2-NBDG-uptake calculated from the 4T1 and 4T07 tumor regions of interest shown in Fig. 3. 2-NBDG-fluorescence in the tumor’s extracellular space reaches a maximum and begins to decay at approximately 7–10 minutes after injection. The rising slope of the curve (indicated by a blue solid line) represents the rate of delivery of 2-NBDG (R_D_) to the tumor. The rate of clearance of 2-NBDG (R_C_) is calculated from the falling slope of the curve. Recovery from hypoxia clearly increases R_D_ (reducing time to maximum) and this is especially apparent in the low-SO_2_ tumor. In both tumors, the falling part of the curve or R_C_ is greater after hypoxia, indicating a slightly faster decay. This leads to lower 2-NBDG_60_ in both low- and high-SO_2_ tumors. Similarly, the R_D_ and R_C_ of the high-SO_2_ 4T07 tumor are higher than the low-SO_2_ tumor at baseline. Breathing hypoxic gas caused a significant increase in R_D_ of 4T1 tumors (**Fig. 6B**; p = 0.002) and R_C_ of the 4T1 (p = 2×10^−4^) and 4T07 tumors (p = 0.03).

Survival curves of R_D_ as a function of SO_2_ reveal the relationship between the two parameters (**Fig. 6C**). At baseline, R_D_ of the 4T1 tumors is lowest for the lowest range of SO_2_ (SO_2_<10%) and increases with SO_2_; however, there are no significant differences in R_D_ at higher SO_2_ ranges. Post hypoxia, there is a large increase in R_D_ as evidenced by the right-shift in the curves (p<0.05). There is also an SO_2_-dependent increase in R_D_ post hypoxia (p<0.05). In the 4T07 tumors, R_D_ of 2-NBDG is greatest at the highest SO_2_ level. Post-hypoxia, no significant differences in R_D_ were observed over the different SO_2_ ranges below the highest SO_2_ value. It is interesting to note that when R_D_ is lowest, the SO_2_ and 2-NBDG_60_ of the 4T1 tumors are also at their lowest. On the other hand, 2-NBDG_60_ of the 4T07 tumors is the lowest when R_D_ and SO_2_ levels are highest (Fig. 4B). These data imply that the R_D_ of 2-NBDG influences uptake in both the 4T1 and 4T07 tumors. However, there does not appear to be a simple relationship between the two parameters. Therefore, the relationship between R_D_, R_C_ and 2-NBDG_60_ for the 4T1 and 4T07 tumors was analyzed next. The values of R_D_ and R_C_ are summarized in **Table 1** for the representative pair of animals in the 4T1 and 4T07 groups.

**Table 1:** Summary of R_D_ and R_C_ values for the representative curves shown in Figure 7A.

		R_D_ (s^−1^)		R_C_ (s^−1^)	
Cell line	SO_2_	Low SO_2_	High SO_2_	Low SO_2_	High SO_2_
4T1	Baseline	0.89	4.37	0.17	0.27
	Post-hypoxia	4.14	7.12	0.21	0.3
4T07	Baseline	2.14	6.58	0.11	0.22
	Post-hypoxia	2.35	4.94	0.19	0.23

### R_D_ in Excess of Glucose Consumption Rate Leads to Lower 2-NBDG_60_

**Figure 7:**
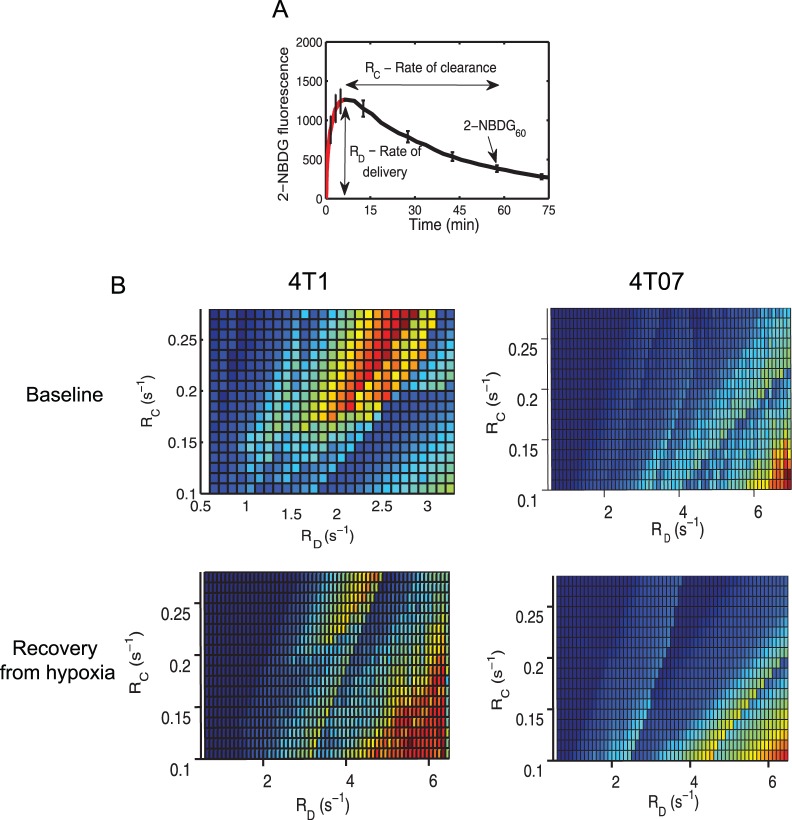
Relationship between SO_2_, R_D_, R_C_ and 2-NBDG_60_ for 4T1 and 4T07 tumors. **A.** Illustration of 2-NBDG uptake curve indicating how R_D_, R_C_ and 2-NBDG_60_ are calculated. **B.** Contour plots showing the relationship between delivery, clearance and uptake of 2-NBDG. R_D_ and R_C_ represent the x- and y-axes, respectively, while 2-NBDG_60_ represents the z-axis projecting out of the x-y plane. For the 4T1 tumors at baseline, 2-NBDG_60_ increases with R_D_ at low values of R_C_. At higher values of R_C_, 2-NBDG_60_ reaches a maximum at R_D_ = 2.5 s^−1^, levels off and then declines gradually for increasing R_D_. After hypoxia, a secondary maximum is seen at very high values of R_D_ (R_D_>6 s^−1^) and low R_C_. In the 4T07 tumors, 2-NBDG_60_ increases with R_D_ and reaches a maximum at approximately 6 s^−1^. The same feature is also present after hypoxia. At higher values of R_C_, 2-NBDG_60_ was nearly negligible for increasing values of R_C_.

**Figure 7A** illustrates how R_D_, R_C_ and 2-NBDG_60_ are derived from the uptake curve of 2-NBDG. **Figure 7B** presents the relationship between R_D_, R_C_ and 2-NBDG_60_. R_D_ and R_C_ represent the x- and y-axes, respectively, while 2-NBDG_60_ represents the z-axis projecting out of the x-y plane. The data shown here are from all tumors within a group. For the 4T1 tumors at baseline, the R_D_ range is 0.5–3 s^−1^. 2-NBDG_60_ is lowest when R_D_ and R_C_ are low (lower left quadrant) and corresponds to poorly oxygenated tumor regions, indicating perfusion-limited uptake. When R_D_ increases, there is a corresponding increase in 2-NBDG_60_ and R_C_, indicating higher uptake due to improved delivery. When R_D_ exceeds approximately 2.5–3 s^−1^, 2-NBDG_60_ plateaus and declines with any further increase in R_D_. This effect is observed clearly at high values of R_C_ (>0.2 s^−1^). The inflection point in the 4T1 tumors at baseline of 2.5–3 s^−1^ is approximately equal to the glucose consumption rates calculated *in vitro*, demonstrating that R_D_ in excess of the glucose consumption rate leads to a decrease in 2-NBDG_60_. After hypoxia, the R_D_ range extends to 6 s^−1^. The 4T1 tumors exhibit a similar decline in 2-NBDG_60_ beyond the glucose consumption rate; however, there is a slight shift in this inflection point to ∼4 s^−1^, suggesting an increase in the glucose consumption rate after breathing hypoxic gas. Due to higher rates of delivery in the 4T07 tumors, regions with R_D_ values <3.5 s^−1^ are mostly absent and the inflection point is not observed. Similar to the 4T1 tumors after hypoxia, a 2-NBDG_60_ maximum exists at high R_D_ (>6 s^−1^) and low R_C_ (<0.15 s^−1^). This is also observed in the 4T07 tumors after hypoxia.

**Table 2** summarizes the interplay seen in the contour plots between the three metabolic endpoints and their relationship to SO_2_. Regions of low 2-NBDG_60_ do not necessarily mean anoxic tissue and delivery limitations; they can also indicate regions of high SO_2_ with delivery rate exceeding the glycolytic rate. Similarly, a high level of 2-NBDG_60_ may not mean hypoxic tissue with great demand; it could potentially indicate tumors demonstrating aerobic glycolysis.

**Table 2:** A summary of possible relationships between NBDG_60_, R_D_, R_C_, and SO_2_ and final outcome corresponding to each combination.

SO_2_	R_D_ (s^−1^)	R_C_ (s^−1^)	2-NBDG_60_	Conclusion/tissue state
Low	Low	Low	Low	Delivery-limited; anoxia
	High	Low	High	High demand; hypoxic
High	High	High	Low	Delivery rate>glycolytic rate
	High	Low	High	Aerobic glycolysis

**Figure 8:**
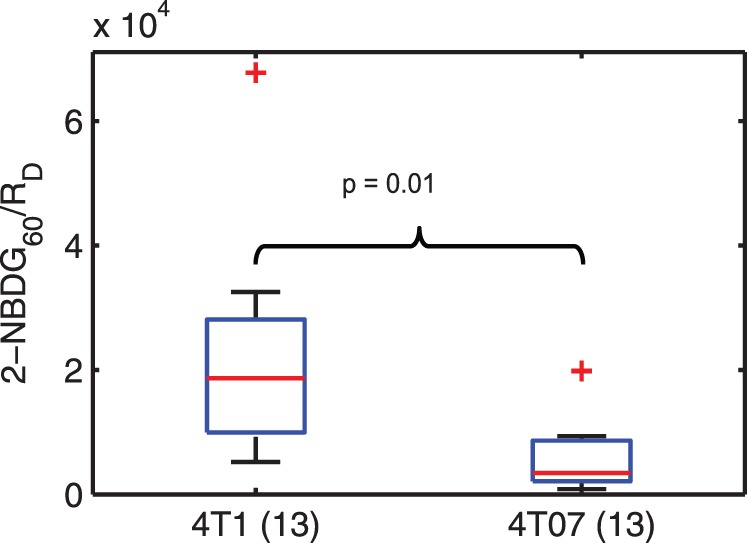
Ratio of 2-NBDG uptake to delivery is higher in 4T1 tumors. The ratio of 2-NBDG_60_ and R_D_, which are indicative of glucose uptake and flow, respectively, is significantly higher in the 4T1 group (p = 0.01) compared to the 4T07 group.

**Figure 8** presents the ratio of 2-NBDG_60_ to R_D_ for combined pre- and post-hypoxia 4T1 and 4T07 tumors. The ratio was significantly higher (n = 13; p = 0.01) in the 4T1 tumors indicating a higher 2-NBDG_60_ and lower R_D_ relative to the 4T07 tumors.

## Discussion

As much as it is important to measure glucose uptake in tumors, it stands to reason that the delivery of contrast agents such as [^18^F]-FDG will be affected by blood flow to the tumor. Therefore, it is important to understand the kinetics of tracer uptake by a tumor in order to understand the underlying implications of the scan. In this paper, we carried out a set of experiments that changed the delivery rate of a glucose tracer - 2-NBDG – to the tumor. Four parameters were established based on optical imaging of the tumor vasculature and uptake of 2-NBDG – vascular oxygenation (SO_2_), rate of delivery of 2-NBDG (R_D_), rate of clearance of 2-NBDG (R_C_), and glucose uptake (2-NBDG_60_). The 60-minute time point was based on overlap between 2-NBDG fluorescence and RFP-positive tumor areas seen in the window chamber and is similar to the time point used in FDG-PET imaging to calculate SUV. We then examined all four endpoints at baseline and after recovery from hypoxia in two tumor lines.

Alternating 1-hour cycles of hypoxia and reoxygenation created a large increase in SO_2_ in the 4T1 tumors that was dependent on baseline SO_2_. The hypoxic or low SO_2_ regions in the 4T1 tumors were absent after breathing hypoxic gas. An increase in blood flow following ischemia is a natural response to a transient oxygen deficit in normal tissue (reactive hyperemia). In this study, the increase in SO_2_ in the 4T1 tumors can be attributed to increased blood flow because the increase in SO_2_ after breathing hypoxic gas was strongly correlated with increased [HbO_2_] and [THb]. This response was not observed in the well-oxygenated 4T07 tumors, presumably due to their high SO_2_ at baseline. Further, there were no changes in vessel diameter or vascular length density in both cell lines following hypoxia. It has been shown previously that the CO_2_ content of gases used in such animal studies could causes changes in tumor blood flow [23], [24]. The gas mixture used in this study was 10% O_2_ - 90% N_2_ and did not contain any CO_2_.

At baseline, the 4T1 and 4T07 tumors revealed contrasting relationships between 2-NBDG_60_ and SO_2_. 4T1 tumors had lowest 2-NBDG_60_ when SO_2_ was lowest. This phenomenon can be explained by perfusion-limited delivery, and hence uptake of 2-NBDG by the tumor. Pre-clinical and clinical studies have shown that lower rates of delivery lead to lower FDG uptake in tumors [7], [25]. In the 4T07 tumors, 2-NBDG_60_ was lowest when SO_2_ was highest. In the absence of any kinetic data, this result would likely have been attributed to a Pasteur-like effect of lower glucose uptake in regions of low hypoxia. However, the kinetic profile of 2-NBDG uptake in the 4T07 tumors demonstrated that there was an SO_2_-dependent increase in R_D_ and that R_D_ was highest in the region where 2-NBDG was lowest. A similar phenomenon has been noticed in untreated primary breast cancers, where Zasadny *et al.* observed a strong correlation between tumor SUV and blood flow but a drop-off in tumor SUV values at high blood flow rates [7]. The authors hypothesized that this was potentially due to the delivery rate exceeding the rate of phosphorylation by hexokinase. The basis for a reduction in 2-NBDG uptake at high flow rates was further established by subjecting the tumors to hypoxia. 2-NBDG_60_ in the 4T1 tumors declined after hypoxia. Again, these results could have been attributed to a Pasteur-like effect where better oxygenation and perfusion leads to a lesser need for 2-NBDG. However, the large increase in flow in the 4T1 tumors led to a SO_2_-dependent increase in R_D_ that was similar to the 4T07 tumors at baseline. In addition, there was no SO_2_-dependent trend in 2-NBDG_60_ in the 4T1 tumors, suggesting that the changes in 2-NBDG_60_ were not a direct result of changes in SO_2_ but rather a result of changes in flow due to better oxygenation.

The contour plots presented in Fig. 7 allow simultaneous visualization of R_D,_ R_C_, and 2-NBDG_60_ and reveal an interesting interplay between the three parameters. At low rates of R_D_ that do not exceed the glucose consumption rate, both 2-NBDG_60_ and R_C_ increase with R_D_. This is feasible because improved delivery is leading to improved uptake by the tumor. At the same time, not all of the delivered 2-NBDG is being taken up, leading to increased clearance. When R_D_ exceeds the glucose consumption rate, 2-NBDG_60_ decreases whereas R_C_ continues to increase. Because more 2-NBDG is being cleared or washed out, 2-NBDG_60_ decreases. Studies of glucose utilization in rat models of ischemic myocardium have revealed a strong dependence on coronary flow and glucose delivery rates. Specifically, glucose extraction as measured by F-FDG increased at lower flow rates and decreased with an increase in flow [26]. After hypoxia, the 4T1 tumors revealed a slight shift in the 2-NBDG_60_ inflection point from 2.5 s^−1^ to 4 s^−1^, suggesting a possible increase in glucose consumption rate after hypoxia. However, both mean 2-NBDG_60_ values as well as the survival curves do not suggest an increase in 2-NBDG_60_ after hypoxia. Recovery from hypoxia extended the range of R_D_ in the 4T1 tumors to reveal a secondary maximum in 2-NBDG_60_ at high R_D_ (>6 s^−1^) and low R_C_ (<0.15 s^−1^). It is interesting to note that the same crest in 2-NBDG_60_ is also observed in the 4T07 tumors at baseline and after hypoxia. Further, the tumor regions corresponding to this 2-NBDG_60_ zone (R_D_>6 and R_C_<0.15 s^−1^) were reasonably oxygenated (mean SO_2_ = 35%), indicating the presence of a potential aerobic glycolysis phenotype common to both cell lines.

These findings suggest that simply measuring glucose tracer uptake at a specific time-point is inadequate; knowledge of tracer kinetics and SO_2_ as well is important to assess the tumor micro-environment. In a clinical study on primary breast tumors, Semple et al. showed that uptake of FDG was affected by blood flow and hence delivery of the glucose tracer [27]. One of their conclusions was that dynamic measurements of FDG uptake that account for the vascular characteristics of the tumor would provide an accurate measure of glucose uptake in tumors. Knowledge of SO_2_ is important because R_D_, which is influenced by the vasculature, does not provide information about SO_2._ Regions of high rates of delivery can be found at different SO_2_ levels, leading to potentially different conclusions (**Table 2**). In addition, accounting for tracer delivery in addition to glucose metabolism has been shown to be of prognostic value in predicting response to therapy. Specifically, the ratio of glucose uptake to blood flow (which affects delivery of tracers) has been proposed as a biomarker of tumor aggression and response to therapy. A low ratio of metabolic rate of FDG uptake to blood flow measured pre-therapy was predictive of better disease-free survival in locally advanced breast cancers treated with neoadjuvant chemotherapy [5], [28]. A high ratio of [^18^F]-FDG uptake to blood flow (measured using [^15^O]H_2_O) has been correlated with tumor aggressiveness and resistance to therapy in pancreatic cancers [29] and poor local control in head and neck cancers [30]. Although our study does not provide direct measures of blood flow, we have shown that our hyperemia protocol increased oxy-hemoglobin and hence R_D_, indicating increased blood flow in the tumor. We used R_D_ and 2-NBDG_60_ to derive a similar ratio of metabolism to blood flow. The ratio of 2-NBDG_60_ to R_D_ of pre- and post-hypoxia tumors combined demonstrated a significantly higher ratio in the 4T1 tumors (Figure 8). It is interesting that a ratio that was found to be higher in therapy-resistant aggressive tumors in clinical studies was similarly higher in the metastatic 4T1 tumors in our study. On the other hand, the 4T07 tumors demonstrated a ratio similar to that of normal tissue in addition to being well oxygenated. A natural follow-up to this study would be to correlate longitudinal measures of endpoints derived in this study with metastatic progression and tumor recurrence in pre-clinical models of breast cancer. Such studies could potentially provide biomarkers to predict long-term outcome in breast tumors at the time of detection.

We are currently developing and validating the endpoints derived in this study using optical spectroscopy, a noninvasive optical fiber-based method. Optical spectroscopy affords the ability to make repeated and noninvasive *in vivo* measurements of tumor morphology and function over a long period of time. The results of this study also hold true for other imaging modalities such as PET in clinical studies and whole animal fluorescence molecular tomography (FMT) that may be used in pre-clinical studies to measure tumor glucose demand in response to therapeutic strategies such as targeted molecular agents or radiation. Targeted therapies such as bevacizumab [31] and PI3K inhibitors [32] have been shown to normalize the vasculature, which can potentially improve tracer delivery to the tumor and thus modulate glucose demand. We have previously demonstrated that radiation can upregulate HIF-1 and hence VEGF, which can stabilize vascular integrity and promote angiogenesis [33], [34]. We have also shown separately that radiation promotes aerobic glycolysis [35]. Thus, therapy can lead to dynamic changes to the tumor vasculature which can influence glucose delivery as well as intrinsic glycolytic demand of tumor cells. Therefore, it is important to separate these effects to accurately determine changes in tumor metabolic demand.

In summary, we have presented a set of optical endpoints that can report on tumor glycolytic demand. Our findings demonstrate that the uptake of glucose tracers such as 2-NBDG is dependent on the rate of delivery to the tumor. Because this delivery rate is influenced by blood flow, it is essential to determine the kinetics of tracer uptake as well as SO_2_ to make informed decisions based on changes in metabolic demand of a tumor. This is especially critical when analyzing tumors before and after events such as chemotherapy and radiation therapy, which are known to induce changes to the tumor microvasculature.
